# A Systematic Review on the Effect of Colchicine in Cardiovascular Disease Management: From Risk Reduction to Comprehensive Care

**DOI:** 10.7759/cureus.97903

**Published:** 2025-11-26

**Authors:** Faysal Azani, Muhammad A Khan, Athar Naveed Yasin, Zill E Huma, Ali Aldujeli, Zahid U Khan

**Affiliations:** 1 Cardiology, St James's Hospital, Dublin, IRL; 2 Cardiology, Salisbury district hospital, Salisbury, GBR; 3 Internal Medicine, Epsom and St Helier University Hospitals NHS Trust, Epsom, GBR; 4 Cardiology, George Eliot Hospital, Nuneaton, GBR; 5 Cardiology, University Hospital Limerick, Limerick, IRL; 6 Cardiology, William Harvey Institute, Queen Mary University of London, London, GBR; 7 Cardiology, University of South Wales, Pontypridd, GBR; 8 Cardiology, University of Buckingham, Buckingham, GBR; 9 Cardiology, Barts Heart Centre, London, GBR

**Keywords:** anti-inflammatory agent, cardiovascular disease (cvd), colchicine, coronary disease, efficacy, heart failure, myocardial infarction, safety, stroke

## Abstract

Inflammation plays an essential role in the pathogenesis of cardiovascular diseases (CVDs), and despite advances in treatments, challenges still exist. Recent studies have explored the use of colchicine in reducing the risk of CVDs. Therefore, the present systematic review aimed to evaluate the impact of colchicine in terms of efficacy, safety, and therapeutic role in managing CVD patients. A comprehensive literature search was performed from different electronic databases, such as PubMed, Scopus, and the Cochrane Library, using keywords associated with the aim of the study, using Preferred Reporting Items for Systematic Reviews and Meta-analyses (PRISMA). Methodological quality assessment was performed using the Cochrane risk of bias-2.0 (RoB-2.0) and the Risk of Bias in Non-Randomized Studies-Intervention (ROBINS-I) tools for randomized controlled trials (RCTs) and non-RCTs, respectively. For the meta-analysis, RevMan 5.4 was used to construct forest plots. Finally, 19 studies were included for qualitative and quantitative analyses. Both male and female patients were included in the studies; however, studies were more skewed toward male patients reported with different types of CVDs, including acute pericarditis, coronary artery disease (CAD), heart failure, and myocardial infarction, and also reported comorbidities, like hypertension (HTN), diabetes, and dyslipidemia. Overall, a low dose of 0.5 mg/daily of colchicine was used. A high adherence rate (>85%) was observed in the studies, with few cases of discontinuation of medications. Numerous studies have reported that colchicine successfully benefits in reducing the inflammatory and other biomarkers, such as C-reactive protein (CRP), Interleukin (IL)-6, and IL-1β. The pooled estimate size for comorbidities, like diabetes, was 0.94, odds ratio (OR) (95% CI, 0.86-1.02, p = 0.13, I^2 ^= 0%). For hypertension and dyslipidemia, it was 1, OR (95% CI, 0.93-1.07, p = 0.95, I^2^ = 9%) and 0.89, OR (95% CI, 0.66-1.2, p = 0.46, I^2^ = 0%), respectively. Meanwhile, the overall effect size was 0.97, OR (95% CI, 0.92-1.02, p = 0.29, I^2^ = 0%). The pooled estimate size for major adverse cardiovascular events (MACEs) and all complications was 0.68, OR (95% CI, 0.36-1.26, p = 0.22, I^2^ = 47%) and 1.60, OR (95% CI, 1.11-2.31, p = 0.01, I^2 ^= 65%). The overall effect size was 1.26, OR (95% CI, 0.92-1.72, p = 0.16, I^2^ = 63%). Furthermore, the pooled estimate size for mortality associated with CVDs and all-cause mortality was 0.77, OR (95% CI, 0.56-1.06, p = 0.11, I^2 ^= 17%) and 1.11, OR (95% CI, 0.72-1.71, p = 0.65, I^2^ = 48%), with the overall effect size of 0.89, OR (95% CI, 0.70-1.12, p = 0.32, I^2 ^= 40%). These outcomes suggest that colchicine offers potential anti-inflammatory benefits in CVD patients, but its clinical impact on outcomes, like MACE and mortality, remains uncertain due to the non-significant difference, warranting further large-scale, high-quality multicenter studies.

## Introduction and background

Colchicine, a drug with medicinal properties, was derived from the medicinal plant *Colchicum autumnale*, and its history as a herbal medicine, according to an Egyptian manuscript (*Eber Papyrus*), goes back at least to the 1500 BCE, while the active ingredient was isolated during the early 1800s and used for the treatment of gout and familial Mediterranean fever [1,2]. In addition, colchicine has also been successfully and effectively applied in immunology, oncology, and dermatology, including epidermolysis bullosa acquistia, aphthous stomatitis, leukocytoclastic vasculitis, and others [3]. Based on the outcomes of clinical trials and meta-analysis, colchicine is recommended for these diseases due to its safety, particularly during pregnancy and lactation [4]. Moreover, due to its anti-inflammatory properties, it has also been used successfully for the treatment of cardiovascular diseases (CVDs), which are the leading cause of morbidity and mortality globally, with conditions such as coronary artery disease (CAD), atherosclerosis, heart failure, peripheral artery disease, myocardial infarction (MI) and ischemic heart disease, placing a significant burden on healthcare systems [1,5]. Despite advancements in lipid-lowering drugs, antiplatelet therapies, antihypertensives, and revascularization procedures, residual inflammatory risk continues to drive adverse cardiovascular clinical outcomes. In this context, colchicine’s unique mechanism of action involves the inhibition of microtubule and subsequent suppression of the interleukin (IL) 1β, IL-6 pathways, and Nod-like receptor protein 3 (NLPR3) inflammasome, making it a promising adjunctive therapy for attenuating vascular inflammation [6,7]. Another important and recognized action of colchicine is the impairment of neutrophil function, which interferes with the production of atherosclerotic plaque. Moreover, it affects the properties of endothelium, macrophages, and smooth muscle cells and interferes with the interaction of platelets and neutrophils [8,9]. It also interferes with inflammatory pathways, like the RhoA/Rho effector kinase (ROCK) pathway, the adhesion and recruitment of neutrophils, the tumor necrosis factor alpha (TNF-α)-induced nuclear factor kappa B (NF-kB), and superoxide production [10]. It further increases the anti-inflammatory cytokines, like transforming growth factor-β (TGF-β) and IL-10, and inhibits pro-IL-18 and messenger RNA expression of caspase-1, which results in the reduction of pro-inflammatory mechanisms, and favorable healing can be promoted [11].

Emerging evidence from published studies suggests that colchicine makes a significant contribution to risk reduction in various cardiovascular contexts, including secondary prevention of stabilization of atherosclerotic plaques, post-MI, and management of atrial fibrillation and pericarditis [12-14]. Most importantly, landmark studies have also highlighted and proved the efficacy of colchicine in reducing composite cardiovascular events [15,16]. Furthermore, a low dose of 0.5 mg/daily significantly reduces the risk of cardiovascular events, particularly among patients with chronic coronary syndrome or acute MI [17]. It also has the potential to be a new standard therapy, which can prevent CAD-associated atherothrombotic events [17]. Even though concerns still exist for long-term safety, particularly in the involvement of gastrointestinal tract [18]. Likewise, studies also reported a non-significant association with the reduction of CVD-associated or all-cause mortality; however, it is correlated with the reduction in the risks for stroke or MI [19]. Meanwhile, in 2004, societies like the European Society of Cardiology recommend it as class I for treating recurrent pericarditis and class IIA for acute pericarditis [20]. In addition, the Food and Drug Administration of the USA approved the administration of low-dose colchicine in June 2023 for the reduction of risk of stroke, MI, coronary revascularization, and deaths due to CVDs [21]. Meanwhile, the American Heart Association/American College of Cardiology recommends colchicine (class IIb) for pericarditis occurring post-MI [22]. Although adverse events such as gastrointestinal problems are associated with the administration of colchicine, a lower dose of 0.5 mg daily for a six-month period had a non-significant impact [23].

Despite its promising outcomes, concerns remain associated with the use of colchicine, as two large randomized trials, LoDoCo2 and COLCOT, had demonstrated a reduction in cardiovascular events, while the COPS trial did not observe any benefits, and its use in the real-world environment is not well described [24]. Moreover, the patient-reported outcomes (such as quality of life) and long-term safety outcomes (gastrointestinal intolerance) are not well reported and necessitate a thorough assessment of the risk-benefit in diverse patient populations worldwide for better comparison, outcomes, and evaluation of the cost-effectiveness. Although another systematic review has previously discussed the impact of colchicine on CVDs, there remains a strong rationale for revisiting and expanding the analysis due to the emergence of new data on long-term efficacy and safety, which warrant an updated synthesis of evidence. Furthermore, the evidence base is still evolving, with ongoing clinical trials aiming to clarify its advantages in broader cardiovascular populations and conditions. Moreover, previously, biomarkers associated with CVDs were not discussed. Given this context, there is a need to gather all evidence-based information, evaluate the consistency of the findings across studies, and provide a comprehensive overview of the effectiveness and safety in CVD management. Therefore, this systematic review and meta-analysis are designed to evaluate and narrate the available evidence on the impact of colchicine on CVD management. This review fills the gaps and will provide real-world data to develop clinical practice and policy recommendations for physicians and decision-makers. The aim of this review was to systematically evaluate and synthesize current evidence on the impact of colchicine in the prevention and management of CVDs, with the following objectives: 1) assessment of the impact of colchicine on major CVD outcomes (e.g., MI, stroke, and mortality), 2) assessment of the impact of colchicine on major cardiovascular events (MACEs) and the biomarkers, and 3) examination of the safety of colchicine in patients with CVDs.

## Review

Methodology

Study Design

This systematic review and meta-analysis were performed in accordance with the 27-item checklist of the Preferred Reporting Items for Systematic Reviews and Meta-analysis (PRISMA) for better transparency and reproducibility of the outcomes [25]. By following PRISMA, we aimed to minimize the selection bias, bring improvement in the methodology, and facilitate the critical appraisal by using the most appropriate risk of bias assessment tools.

Search Strategy

For the retrieval of the most relevant studies, a literature search was performed from different electronic databases, such as PubMed, the Cochrane Library, and Scopus, using specific keywords and search terms like “colchicine” OR “anti-inflammatory therapy” AND “cardiovascular disease” OR “CVD” OR “coronary artery disease” OR “CAD” OR “myocarditis” OR “pericarditis” OR “major adverse cardiovascular events” OR “MACE” OR “biomarkers.” More appropriate modifications were made for each database (Table 1).

**Table 1:** Literature search from different databases.

Databases	Search terms
PubMed	(("colchicine"[All Fields] OR "anti-inflammatory therapy"[All Fields]) AND ("cardiovascular diseases"[MeSH Terms] OR "CVD"[All Fields] OR "coronary artery disease"[MeSH Terms] OR "CAD"[All Fields] OR "myocarditis"[MeSH Terms] OR "pericarditis"[MeSH Terms] OR "major adverse cardiovascular events"[All Fields] OR "MACE"[All Fields] OR "biomarkers"[MeSH Terms]))
Cochrane Library	((“colchicine” OR “anti-inflammatory therapy”)):ti,ab,kw AND ((“cardiovascular disease” OR “CVD” OR “coronary artery disease” OR “CAD” OR “myocarditis” OR “pericarditis” OR “major adverse cardiovascular events” OR “MACE” OR “biomarkers”)):ti,ab,kw
Scopus	(“colchicine” OR “anti-inflammatory therapy”) AND (“cardiovascular disease” OR “CVD” OR “coronary artery disease” OR “CAD” OR “myocarditis” OR “pericarditis” OR “major adverse cardiovascular events” OR “MACE” OR “biomarkers")

Inclusion Criteria

The population, intervention, control/comparator, outcomes (PICO) framework was used to include the studies in the present review (Table 2).

**Table 2:** Detailed description of the PICO framework.

PICO component	Description
Population (P)	Adult patients aged >18 years with any form of cardiovascular disease (CVD), including ischemic heart disease, acute coronary syndrome, atherosclerosis, myocardial infarction, heart failure, and stroke
Intervention (I)	Colchicine administration as monotherapy or in combination with other standard therapies
Comparison (C)	Standard treatment without colchicine or placebo
Outcomes (O)	Primary outcomes: reduction in CVDs and major cardiovascular events (MACEs). Secondary outcomes: examining novel biomarkers (IL-6, IL-1β), pericarditis resolution/CVDs occurrence, and complications

Eligible studies included randomized controlled trials (RCTs) and non-RCTs with comparison groups, such as observational, retrospective, and cohort studies. Only studies published in English were considered.

Exclusion Criteria

Similarly, certain exclusion criteria were set for excluding studies, like studies that included paediatric patients (<18 years), non-cardiovascular studies (e.g., gout-only trials). Studies without control groups, studies that do not report the primary and secondary outcomes of the present study, and reviews, case studies, case series and literature reviews, editorials, abstracts, commentary, letters to editors, proceeding papers, and studies published in non-English languages were excluded.

Study Screening and Selection Process

**Figure 1:**
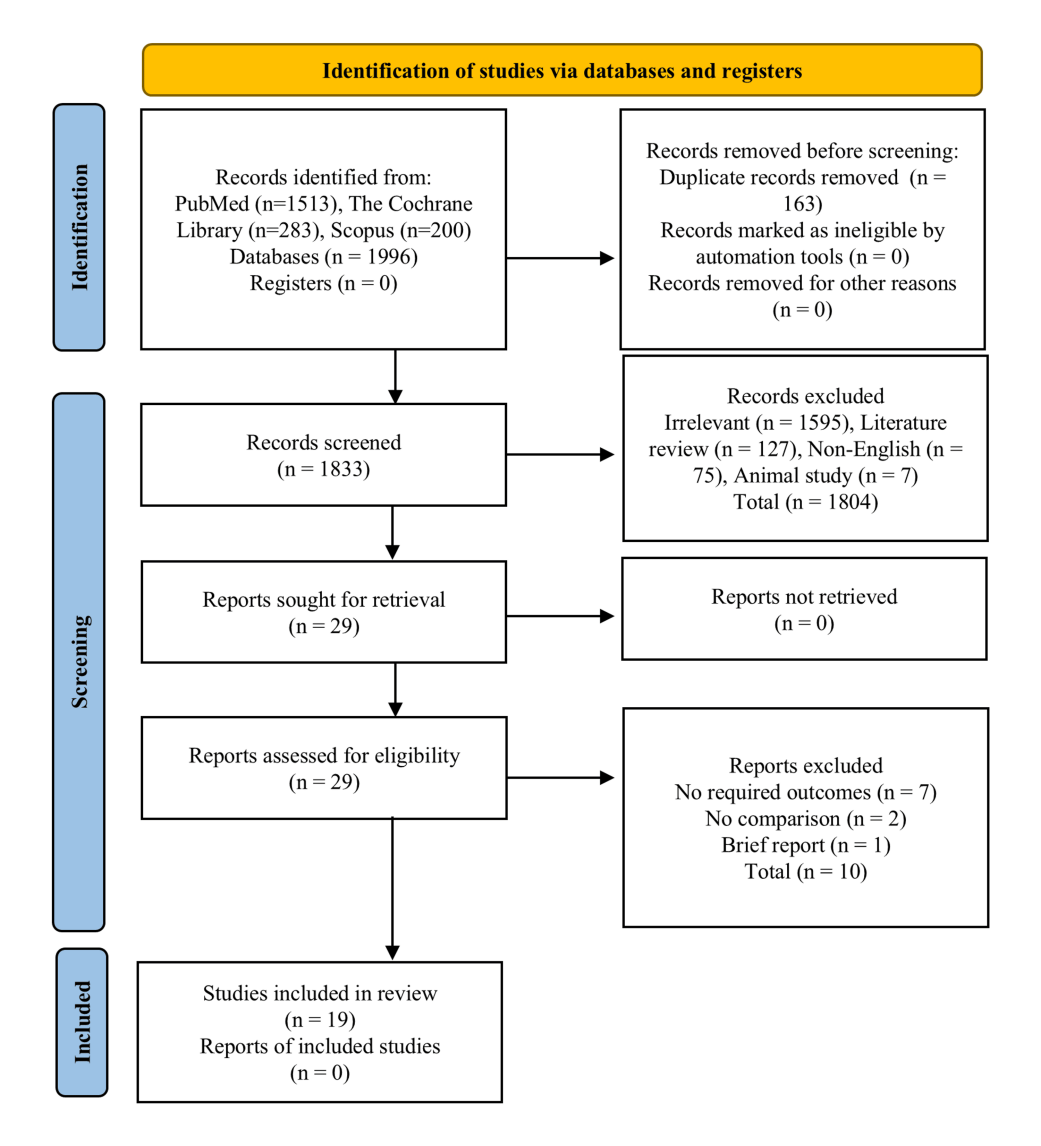
Preferred Reporting Items for Systematic Reviews and Meta-analysis (PRISMA) flow chart for the selection of studies.

For the screening and selection of studies, a four-stage PRISMA flow chart was used. In the first stage, 1,996 studies were retrieved from different electronic databases, the library was imported into EndNote X9 referencing software, and 163 duplicates were removed. In the second stage, 1,833 studies were screened as per the titles and abstracts, 29 studies with relevance were moved to the third stage, and the remaining 1,804 irrelevant studies were excluded due to the following reasons (irrelevant = 1,595, literature reviews = 127, non-English = 75, and animal studies = 7). The full-text assessment was performed in the third stage, and strict inclusion/exclusion criteria were followed. Nineteen selected studies were moved to the final stage for further qualitative and quantitative analysis. To minimise selection bias, two independent reviewers screened all titles, abstracts, and full texts. Any disagreements were resolved through discussion or with the involvement of a third reviewer. The remaining 10 studies were excluded for reasons such as studies that had no required outcomes, no comparison, or a brief report (Figure 1).

Data Extraction

A predefined data extraction form was used for the extraction of all relevant information, which included different categories: study characteristics (author ID, country, sample size, dosage, and follow-up duration), patient characteristics (age and gender), outcomes (MACEs, biomarker changes (e.g., IL-6, CRP), and pericarditis resolution), patient-centered metrics (quality of life, adherence, and adverse events), and subgroup analyses (comorbidities, MACEs, all complications, CVD-associated mortality, and all-cause mortality).

Methodological Quality Assessment

The Cochrane Collaboration risk of bias (RoB)-2.0 assessment tool was used for the methodological quality assessment of each RCT study. Each study was evaluated in the domain of randomization process, deviation from intended intervention, missing outcome data, measurement of the outcomes, and selection of reported results. After evaluation, each study was categorized as low RoB, high RoB, and some concerns. For non-RCT studies, the Risk of Bias in Non-Randomized Studies-Intervention (ROBINS-I) assessment tool was used, and responses were categorized as low RoB, high RoB, and some concerns [26]. For visualization of the assessment outcomes, robvis, a web-based tool, was used [27].

Certainty of Evidence

The Grading of Recommendations, Assessment, Development and Evaluation (GRADE) framework was used for the certainty of evidence. Later, each outcome was rated with very low, low, moderate, and high certainty of evidence [28].

Meta-Analysis

Qualitative data were synthesized in table form, while the quantitative data were analyzed using RevMan 5.4 (Cochrane, London, UK). Continuous data with the same scale for the outcomes for intervention and control/comparator (sample size and events) were used for the construction of forest plots for the overall effect size. Sub-group analyses were performed in the case of MACE, all complications, CVD-associated mortality rate, and all-cause mortality rate. In addition, heterogeneity was assessed by using I2 statistics, tau-squared (T2), and X2 Cochrane Q tests. The I2 statistics were divided into four categories, i.e., 0-29%, 30-49%, 50-74%, and 75-100%, and were interpreted as low, moderate, substantial, and very high heterogeneity, respectively. In case of no heterogeneity, a fixed effect model was used, and when there was low, moderate, or high heterogeneity, a random effect model was used. Likewise, a value of T2 > 1 indicated inter-study variability, and the Q test measured the variation around the weighted means. All the analyses were performed using a random effect model and considered significant at a p-value of 0.05 [29,30]. For publication bias, a funnel plot was constructed.

Results

General Characteristics

Most of the studies were published in 2019 [31-34], followed by three studies in 2013, 2020, and 2021. Moreover, the majority of the studies were reported from Australia [33,35-37], followed by Canada [15,38], the USA [32,39], France [40,41], Greece [42,43], Italy [44,45], Iran [46], Japan [34], Spain [31], the Netherlands [6], and China [47]. A varied number of sample sizes were used, with a maximum of 2,762 and 2,760 in the intervention and control groups, respectively [38]. Meanwhile, nine and 11 were the minimum number of patients in the intervention and control groups, respectively [35]. Both male and female patients were included in the studies; however, studies were more skewed toward male patients (Table 2). In terms of age, overall, elderly patients were included in the intervention and control groups, with a maximum of 69.80 years in the intervention group and 67.55 years in the control group [35]. The minimum age was 43.8 and 44.8 for the intervention and control groups, respectively [31]. Patients were reported with different types of CVDs, with comorbidities like hypertension, diabetes, and dyslipidemia described in Table 3.

**Table 3:** Summary of the general characteristics of the included studies. HTN: hypertension, RCT: randomized controlled trial, NA: not available, M: male, F: female

Study ID	Country	Study design	Sample size	Gender (M: F)	Age	CVD type	Comorbidities
Deftereos et al. [43]	Greece	RCT	Intervention group = 100, Control group = 96	Intervention group = 63:37, Control group = 65:31	Intervention group = 63.7, Control group = 63.5	Bare-metal stent restenosis	HTN, diabetes
Imazio et al. [45]	Italy	RCT	Intervention group = 120, Control group = 120	Intervention group = 71:49, Control group = 74:46	Intervention group = 53.5, Control group = 50.7	Acute pericarditis	NA
Nidorf et al. [15]	Canada	RCT	Intervention group = 282, Control group = 250	Intervention group = 251:31, Control group = 222:28	Intervention group = 66, Control group = 67	Coronary diseases	Diabetes
Imazio et al. [44]	Italy	RCT	Intervention group = 120, Control group = 120	Intervention group = 66:54, Control group = 54:66	Intervention group = 48.6, Control group = 48.9	Acute pericarditis	NA
(Deftereos et al. [42]	Greece	RCT	Intervention group = 140, Control group = 139	Intervention group = 94:46, Control group = 93:46	Intervention group = 66.9, Control group = 66.4	Chronic heart failure	HTN, diabetes, dyslipidemia
Robertson et al. [35]	Australia	RCT	Intervention group = 9, Control group = 11	Intervention group = 7:2, Control group = 8:3	Intervention group = 69.80, Control group = 67.55	Acute coronary disease	HTN, diabetes, dyslipidemia
Akodad et al. [41]	France	RCT	Intervention group = 23, Control group = 21	Intervention group = 19:4, Control group = 16:5	Intervention group = 60.1, Control group = 59.7	Acute myocardial infarction	HTN, diabetes, dyslipidemia
Vaidya et al. [36]	Australia	Observational study	Intervention group = 40, Control group = 40	Intervention group = 32:8, Control group = 30:10	Intervention group = 56.3, Control group = 58.4	Acute coronary disease	HTN, diabetes, dyslipidemia
Hennessy et al. [33]	Australia	RCT	Intervention group = 119, Control group = 118	Intervention group = 89:30, Control group = 93:25	Intervention group = 61, Control group = 61	Acute myocardial infarction	HTN, diabetes
Kajikawa et al. [34]	Japan	RCT	Intervention group = 14, Control group = 14	27:1	68	Coronary disease	HTN, diabetes
Sambola et al. [31]	Spain	RCT	Intervention group = 59, Control group = 51	Intervention group = 48:11, Control group = 44:7	Intervention group = 43.8, Control group = 44.8	Acute idiopathic pericarditis	NA
Tardif et al. [32]	USA	RCT	Intervention group = 2366, Control group = 2379	Intervention group = 1894:472, Control group = 1492:437	Intervention group = 60.6, Control group = 60.5	Myocardial infarction	HTN, diabetes
Nidorf et al. [38]	Canada	RCT	Intervention group = 2762, Control group = 2760	Intervention group = 2305:457, Control group = 2401:389	Intervention group = 65.8, Control group = 65.9	Chronic coronary disease	HTN, diabetes
Shah et al. [39]	USA	RCT	Intervention group = 206, Control group = 194	Intervention group = 193:13, Control group = 181:13	Intervention group = 65.9, Control group = 66.6	Patients underwent percutaneous coronary intervention	HTN, diabetes, dyslipidemia
Tong et al. [37]	Australia	RCT	Intervention group = 396, Control group = 399	Intervention group = 322:74, Control group = 310:89	Intervention group = 59.7, Control group = 60	Coronary disease	HTN, diabetes
Akrami et al. [46]	Iran	RCT	Intervention group = 120, Control group = 129	Intervention group = 86:34, Control group = 87:42	Intervention group = 56.9, Control group = 56.89	Acute coronary disease	HTN, diabetes
Mewton et al. [40]	France	RCT	Intervention group = 101, Control group = 91	Intervention group = 80:21, Control group = 74:17	Intervention group = 59, Control group = 60.9	Acute myocardial infarction	HTN, diabetes, dyslipidemia
Opstal et al. [6]	The Netherlands	RCT	Intervention group = 2762, Control group = 2760	NA	NA	Chronic coronary disease	HTN, diabetes
Pan et al. [47]	China	RCT	Intervention group = 59, Control group = 62	Intervention group = 31:28, Control group = 39:23	Intervention group = 58.90, Control group = 59.92	Post-myocardial infarction and chronic coronary disease	HTN, diabetes, stroke, chronic kidney diseases

Intervention Characteristics

Most of the studies used colchicine with standard or conventional care; however, certain studies used colchicine as a monotherapy for the treatment of CVDs [32,35,36,39,40,42,43,47]. Overall, a low dose of 0.5 mg/daily of colchicine was used. Few studies used 0.5 mg/twice/day or 1 mg/daily [31,35,41,45]. The duration of administration of colchicine varied and ranged from two days to 36 months [15,35], and patients were also followed for a maximum of 36 months (Table 2). The outcomes of the intervention were compared with the standard care or conventional treatment or placebo, as indicated in Table 2. Meanwhile, a high adherence rate (>85%) was observed in the studies, with few cases of discontinuation of medications [15,31,36,37]. Biomarkers, which were assessed more frequently, included IL-6, WBC, hs-CRP, IL-1β, fibrinogen, neutrophil, and procalcitonin (Table 4).

**Table 4:** Summary of characteristics of intervention and biomarkers assessed. CRP: C-reactive protein, IL-6: interleukin-6, hs-CRP: high-sensitivity C-reactive protein, WBC: white blood cells, NA: not available, mg: milligram

Study ID	Intervention (mono or in combination)	Dosage	Duration (months)	Control	Adherence	Follow-up (months)	Biomarkers assessed
Deftereos et al. [43]	Colchicine	0.5 mg/daily	6	Placebo	95.60%	6	NA
Imazio et al. [45]	Colchicine + conventional treatment	0.5-1 mg/daily	3	Placebo + conventional treatment	>95%	22	WBC, CRP
Nidorf et al. [15]	Colchicine + conventional treatment	0.5 mg/daily	36	Conventional treatment	Early withdrawal:30, Late withdrawal: 32	36	NA
Imazio et al. [44]	Colchicine + conventional anti-inflammatory treatment	0.5 mg/twice daily	6	Placebo + conventional treatment	>95%	20	CRP
(Deftereos et al. [42]	Colchicine	0.5 mg/twice daily	6	Placebo	Intervention group=91%, Control group=97%	6	CRP, IL-6
Robertson et al. [35]	Colchicine	1 mg followed 0.5 mg after 1 hour/daily	2 days	No treatment	NA	NA	IL-18, IL-1β, IL-6
Akodad et al. [41]	Colchicine + optimal medical treatment	1 mg/daily	1	Optimal medical treatment	20/23=86.95%	1	Procalcitonin, leukocyte, hs-cTnT, CRP
Vaidya et al. [36]	Colchicine	0.5 mg/daily	NA	Placebo	4/40=10% discontinuation	12.6	hs-CRP
Hennessy et al. [33]	Colchicine + standard care	0.5 mg/daily	1	Placebo + standard care	2% vs 4% of patients lost follow-up	1	CRP, IL-6, WBC, Neutrophil, Lymphocyte
Kajikawa et al. [34]	Colchicine + conventional treatment	0.5 mg/daily	7 days	Placebo + conventional treatment	NA	14 days	hs-CRP
Sambola et al. [31]	Colchicine + conventional anti-inflammatory treatment	1-0.5 mg/12 hours	3	Conventional anti-inflammatory treatment	7 patients discontinued	30.2	NA
Tardif et al. [32]	Colchicine	0.5/daily	NA	Placebo	NA	22.6	NA
Nidorf et al. [38]	Colchicine + conventional treatment	0.5 mg/daily	NA	Placebo + conventional treatment	89.50%	28.6	NA
Shah et al. [39]	Colchicine	0.6 mg	NA	Placebo	NA	1	hs-CRP, IL-6
Tong et al. [37]	Colchicine + conventional treatment	1^st^ month: 0.5 mg/twice daily; Eleven months: 0.5 mg/daily	11	Placebo + conventional treatment	15% patients discontinued medication	12	NA
Akrami et al. [46]	Colchicine + standard treatment	0.6/daily	6	Placebo + standard treatment	NA	6	WBC, Lymphocyte, Neutrophil
Mewton et al. [40]	Colchicine	0.5/twice daily	5 days	Placebo	NA	3	WBC, Neutrophil, Fibrinogen, CRP
Opstal et al. [6]	Colchicine + conventional treatment	0.5 mg/daily	NA	Placebo + conventional treatment	NA	28.6	NA
Pan et al. [47]	Colchicine	0.5 mg/daily	Before operation: 3 days; After operation: 5 days	Placebo	NA	5 days	Procalcitonin, IL-6, CRP

Numerous studies have reported that colchicine successfully benefits in reducing the inflammatory and other biomarkers, such as CRP, IL-6, and IL-1β [34-36,39,42,47]; however, certain studies also reported non-significant differences and found no impact of colchicine on the biomarkers [33,40,46]. Furthermore, a lower rate of MACE and mortality was reported after the intervention, with some studies reporting a non-significant difference between intervention and control groups [6,33,39]. Colchicine also successfully lowered the rate of pericarditis and CVD events [6,15,32,37,38,44]. In terms of complications, gastrointestinal side effects or adverse effects were the most frequent complications identified in most of the studies, and the conclusion of each study is presented in Table 5.

**Table 5:** Summary of outcomes (MACEs, mortality, and safety profile). MACE: major adverse cardiovascular events, CRP: C-reactive protein, hs-CRP: high-sensitivity C-reactive protein, CVD: cardiovascular disease, PCI: percutaneous coronary intervention, NA: not available, g: gram, mg: milligram, ng: nanogram, L: liter, dL: decilitre, pg/ml: picogram/milliliter

Study ID	MACEs	Mortality due to CVDs	Biomarker changes	Pericarditis resolution/occurrence of CVDs	Complications	Conclusion
Deftereos et al. [43]	NA	NA	NA	NA	Gastrointestinal involvement	Colchicine is associated with less neointimal hyperplasia and decreased in-stent restenosis.
Imazio et al. [45]	NA	NA	NA	One case in the control group showed constrictive pericarditis.	Gastrointestinal involvement	Colchicine in combination significantly reduced the rate of incessant or recurrent pericarditis.
Nidorf et al. [15]	NA	Interventional group = 4, Control group = 10	NA	5.3% vs. 16% occurrence of CVDs	Gastrointestinal involvement	Effective when used in combination with conventional treatment
Imazio et al. [44]	NA	NA	NA	21.6% vs. 42.5% recurrent pericarditis	Gastrointestinal involvement	Colchicine significantly reduced the rate of subsequent recurrences of pericarditis.
Deftereos et al. [42]	NA	NA	CRP and IL-6 levels were significantly reduced in the intervention group (p < 0.001).	NA	Gastrointestinal	Effective in reducing biomarkers, however, did not impact functional outcomes
Robertson et al. [35]	NA	NA	IL-18 and IL-1β were similar between groups.	NA	NA	Inhibits inflammatory biomarkers
Akodad et al. [41]	After one month, 2.2% in each group (p = 1)	Intervention group = 0, Control group = 0	Procalcitonin: 0.48 vs. 0.11 mg/dL (p = 0.38), leukocyte: 13.1 vs. 11.5 g/L (p = 0.16), hs-cTnT: 6,476.774 vs. 4,635.581 ng/L (p = 0.14), CRP: 29.03 vs. 21.86 mg/L (p = 0.38)	NA	Gastrointestinal involvement; however, only three patients discontinued the treatment.	Colchicine could not be demonstrated, with no improvement in inflammatory profiles or diminution of infarct size.
Vaidya et al. [36]	2 vs. 1	Intervention group = 0, Control group = 0	Hs-CRP: 1.85 vs. 2.26 mg/L	NA	Gastrointestinal involvement	Colchicine therapy favorably modifies the coronary plaque.
Hennessy et al. [33]	After one month, 16% vs. 18% in each group (p = 0.85)	Intervention group = 0, Control group = 0	Non-significant changes in CRP, IL-6, WBC, neutrophil, and lymphocyte	NA	Gastrointestinal involvement	Colchicine did not reduce CRP levels significantly.
Kajikawa et al. [34]	NA	NA	hs-CRP level was significantly (p = 0.003) reduced in the intervention group.	NA	No serious adverse events	Colchicine did not improve endothelial function.
Sambola et al. [31]	No MACE	NA	NA	Non-significant difference in recurrent pericarditis	Gastrointestinal involvement	Colchicine did not impact the recurrence rate.
Tardif et al. [32]	NA	Intervention group = 20, Control group = 24	NA	Lower risk of ischemic cardiovascular events	Gastrointestinal involvement, pneumonia	Colchicine significantly lowers the risk of ischemic cardiovascular events than placebo.
Nidorf et al. [38]	Occurred	Intervention group = 187, Control group = 264	NA	2.5 vs. 3.6 rate for disease occurrence	Gastrointestinal involvement, infection, pneumonia	Cardiovascular events are significantly lower in the intervention group.
Shah et al. [39]	11.7% vs. 12.9% (p > 0.05)	Intervention group = 1, Control group = 1	Significantly reduced hs-CRP 11% vs. 66% (p = 0.001)	NA	Intraprocedural complications	Colchicine attenuated the increase in IL-6 and hsCRP concentrations after PCI.
Tong et al. [37]	NA	Intervention group = 8, Control group = 1	NA	5% vs. 9.5%, p < 0.05	Gastrointestinal involvement	Non-significant impact on the CVD outcomes and had higher mortality
Akrami et al. [46]	After six months, lower rate in the intervention group (p = 0.001)	Intervention group = 4, Control = 2	Non-significant changes in WBC, lymphocyte, and neutrophil	NA	Gastrointestinal involvement	Colchicine significantly reduces MACEs and improves the survival rate.
Mewton et al. [40]	NA	NA	Non-significant difference	Non-significant difference in the infarct size	Gastrointestinal involvement	No significant impact on the infarct size
Opstal et al. [6]	No significant difference	Non-significant difference	NA	2.4 vs. 3.3 events per 100 person/years	NA	Effective
Pan et al. [47]	No MACE	Intervention group = 0, Control group = 0	IL-6: 14.86 vs. 18 pg/mL (p = 0.08); procalcitonin: 0.25 vs. 0.68 ng/mL (p < 0.0001); CRP: 38.31 vs. 40.04 mg/L (p = 0.56)	No constrictive pericarditis	Gastrointestinal involvement	Low-dose colchicine for a short period was effective in attenuating the postoperative biomarkers of inflammation and myocardial injury.

Meta-Analysis: Subgroup Analysis

**Figure 2:**
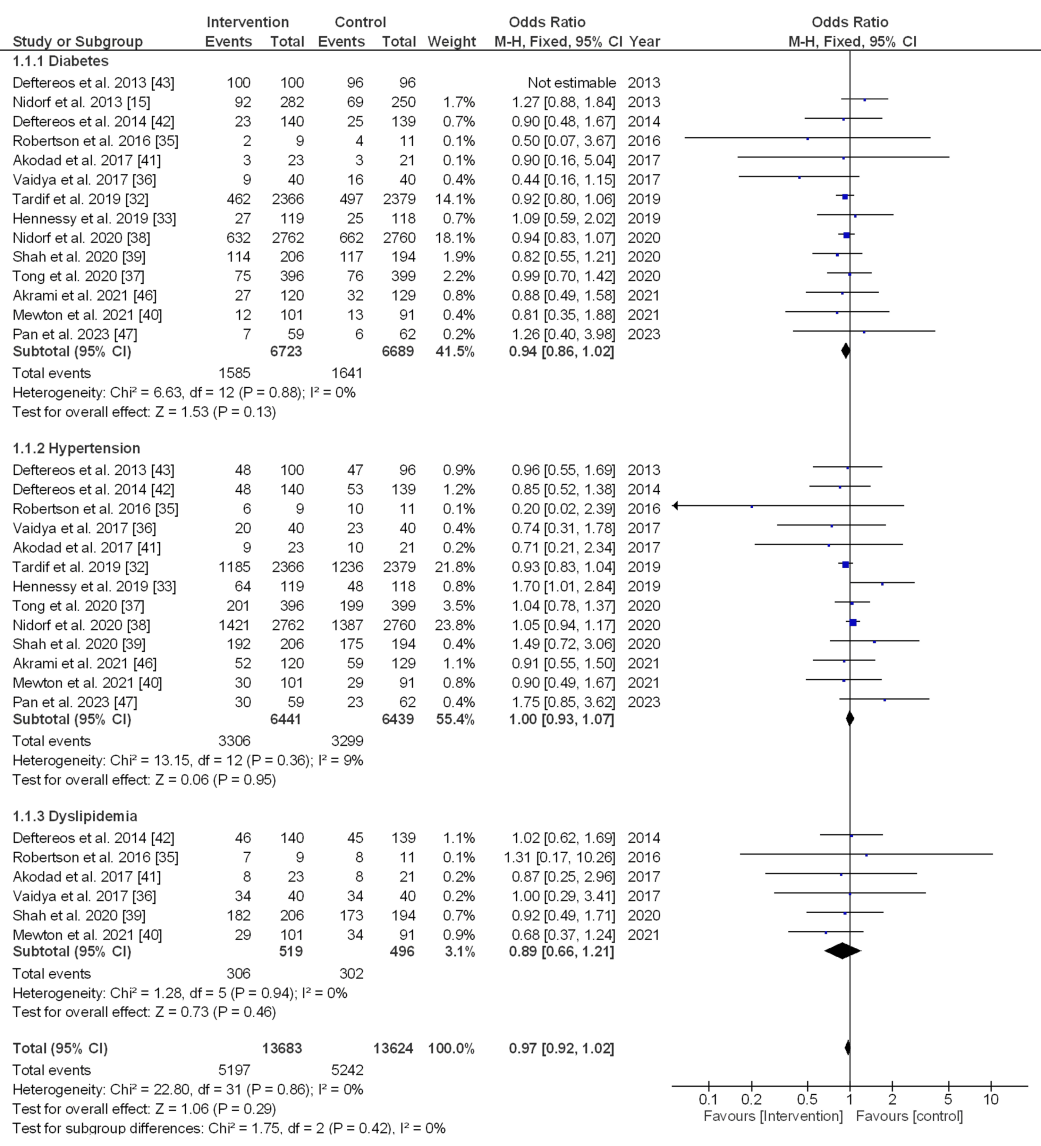
Forest plot (fixed effect model) for the subgroup analysis (comorbidities) between the intervention and control groups.

Comorbidities: The pooled estimate size of 14 studies for diabetes was 0.94, odds ratio (OR) (95% CI, 0.86-1.02), with a non-significant difference (p = 0.13) and low heterogeneity (I_2_ = 0%). Likewise, for hypertension, the pooled estimate of 13 studies was 1, OR (95% CI, 0.93-1.07), with a non-significant difference (p = 0.95) and low heterogeneity (I_2_ = 9%). For dyslipidemia, the pooled effect size of six studies was 0.89, OR (95% CI, 0.66-1.21), with a non-significant difference (p = 0.46) and low heterogeneity (I_2 _= 0%). Meanwhile, the overall effect size was 0.97, OR (95% CI, 0.92-1.02), with non-significant differences (p = 0.29) and low heterogeneity (I_2_ = 0%), as indicated in Figure 2.

**Figure 3:**
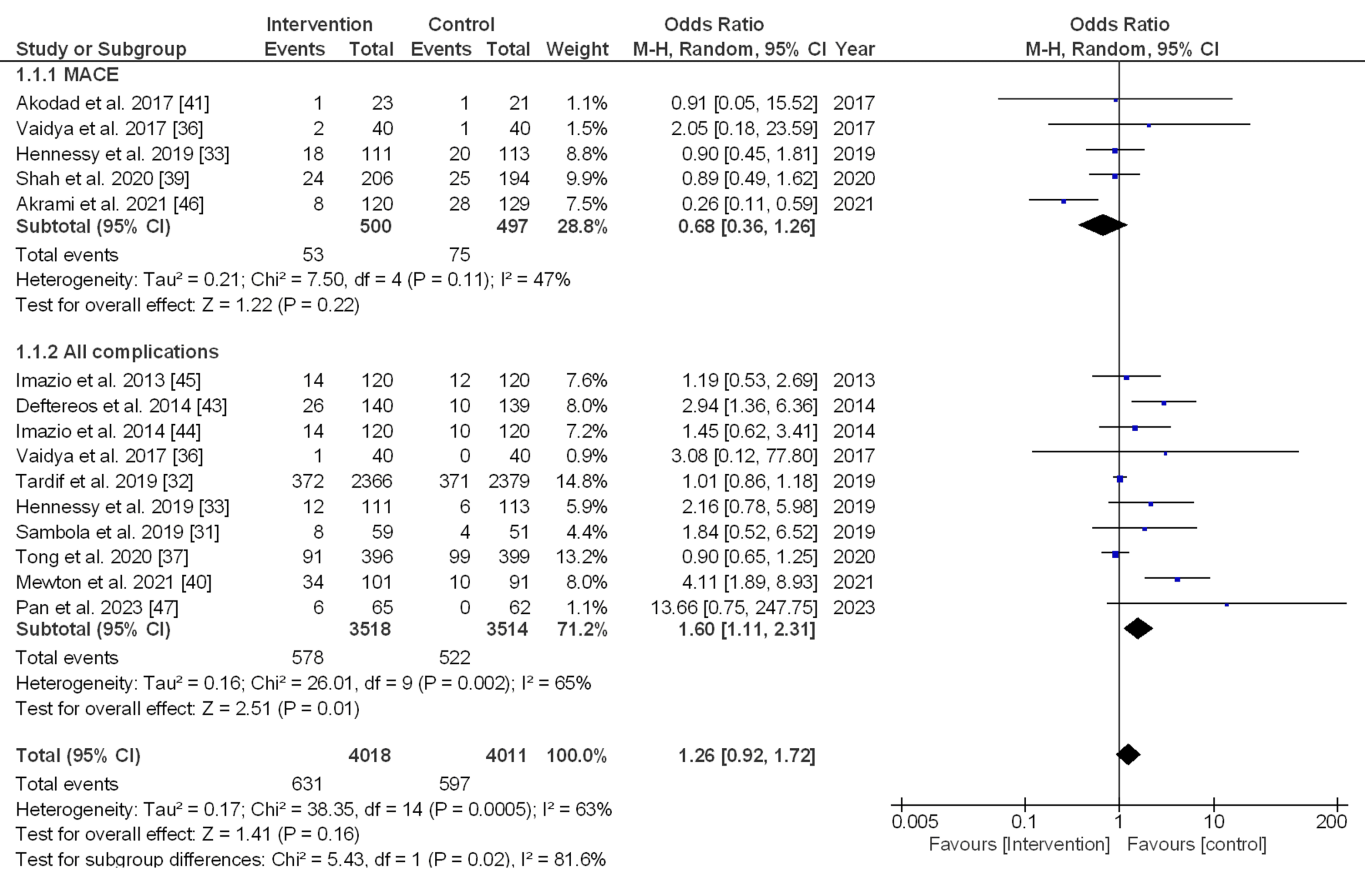
Forest plot (random effect model) for the subgroup analysis (MACE vs. all complications) between the intervention and control groups. MACE: major adverse cardiovascular event

MACEs versus all complications: The pooled estimate size of five studies for MACEs was 0.68, OR (95% CI, 0.36-1.26), with a non-significant difference (p = 0.22) and moderate heterogeneity (I_2_ = 47%). For all complications, the pooled effect size of 10 studies was 1.60, OR (95% CI, 1.11-2.31), with a significant difference (p = 0.01) and a substantial heterogeneity (I_2_ = 65%). Meanwhile, the overall effect size was 1.26, OR (95% CI, 0.92-1.72), with a non-significant difference (p = 0.16) and a substantial heterogeneity (I_2_ = 63%), as described in Figure 3.

**Figure 4:**
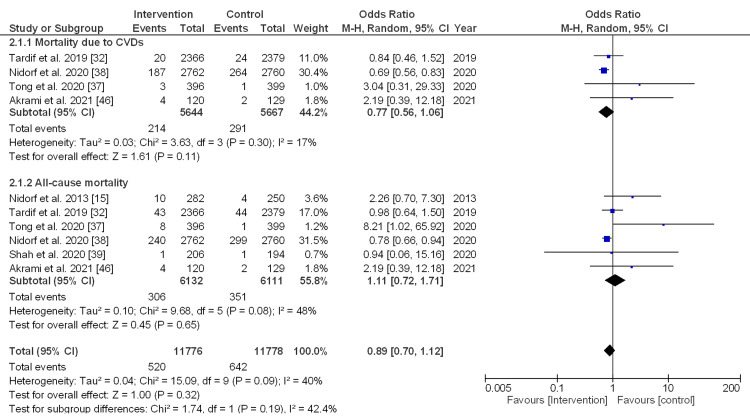
Forest plot (random effect model) for the subgroup analysis (CVD-associated mortality vs. all-cause mortality) between the intervention and control groups. CVD: cardiovascular disease

CVD-associated mortality vs. all-cause mortality: The pooled estimate size of four studies for mortality associated with CVDs was 0.77, OR (95% CI, 0.56-1.06), with a non-significant difference (p = 0.11) and low heterogeneity (I_2_ = 17%). For all-cause mortality, the pooled effect size of six studies was 1.11, OR (95% CI, 0.72-1.71), with a non-significant difference (p = 0.65) and moderate heterogeneity (I_2_ = 48%). Meanwhile, the overall effect size was 0.89, OR (95% CI, 0.70-1.12), with non-significant differences (p = 0.32) and moderate heterogeneity (I_2_ = 40%), as described in Figure 4.

**Figure 5:**
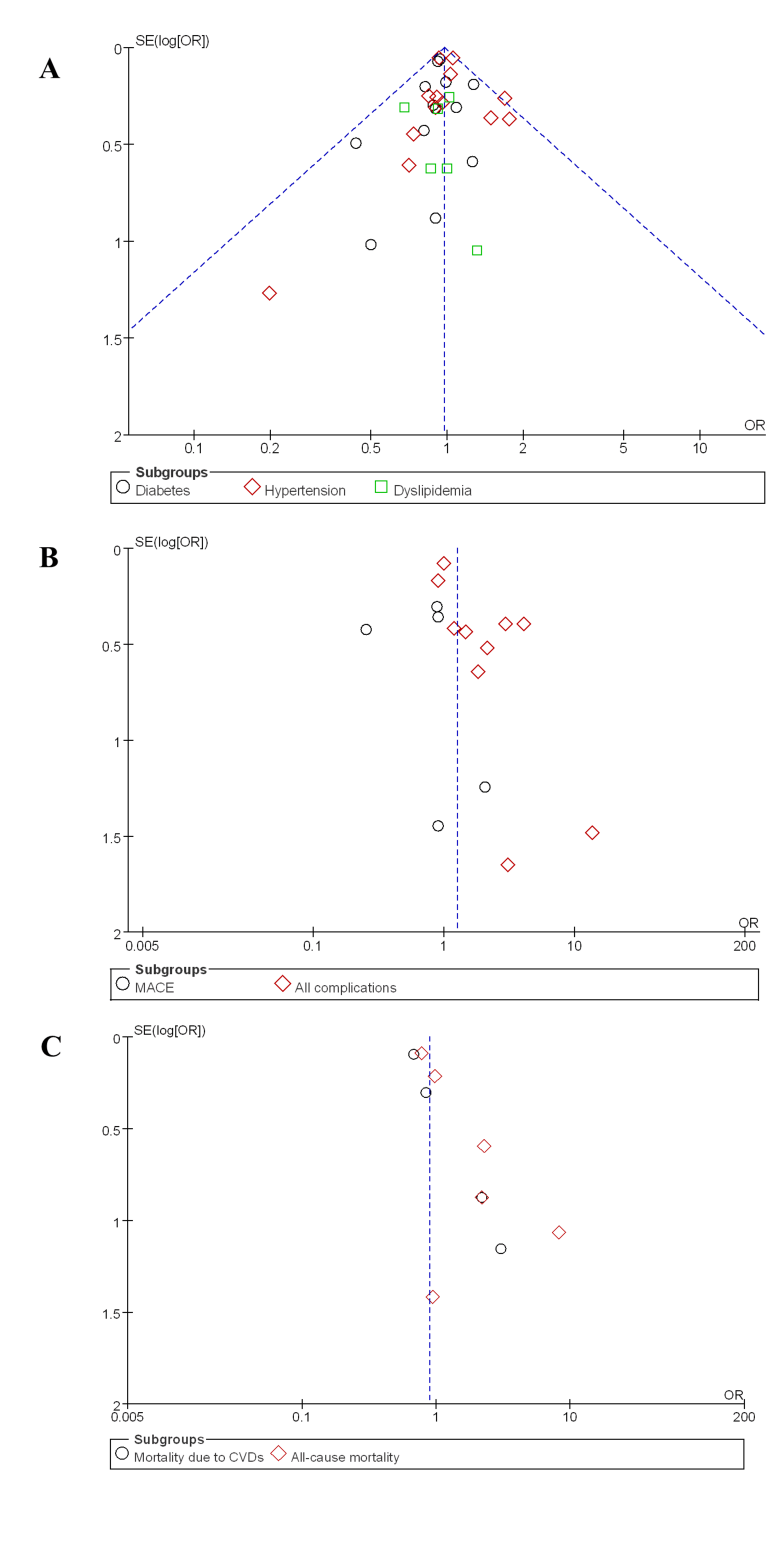
Publication bias among studies. A: Publication bias among studies addressing comorbidities and clearly making a funnel shape, suggesting no publication bias. B: Studies reported outcomes, like MACEs and all complications, and showed no publication bias. C: This figure demonstrated that studies addressing CVD-associated mortality and all-cause mortality had no publication bias. MACE: major adverse cardiovascular event, CVD: cardiovascular disease

Publication bias: Overall, no publication bias was observed among the studies when compared to the intervention and control groups in terms of comorbidities, as studies were equally distributed around the effect size line (Figure 5A). Likewise, no publication was observed among studies addressing MACE versus all complications, as studies were distributed around the effect size line (Figure 5B). Similarly, studies focusing on CVD-associated mortality versus all-cause mortality also demonstrated no publication bias. Studies were found around the symmetrical line and present on both sides of the line, clearly making a funnel shape (Figure 5C). This suggests that the studies included in the present systematic review and meta-analysis were well-balanced and not selectively reported to favour significant or positive outcomes. This symmetry indicates that smaller studies with both low and high effect sizes were equally reported, reducing the likelihood that outcomes were influenced by the selective publication of only favourable findings.

Methodological Quality Assessment

**Figure 6:**
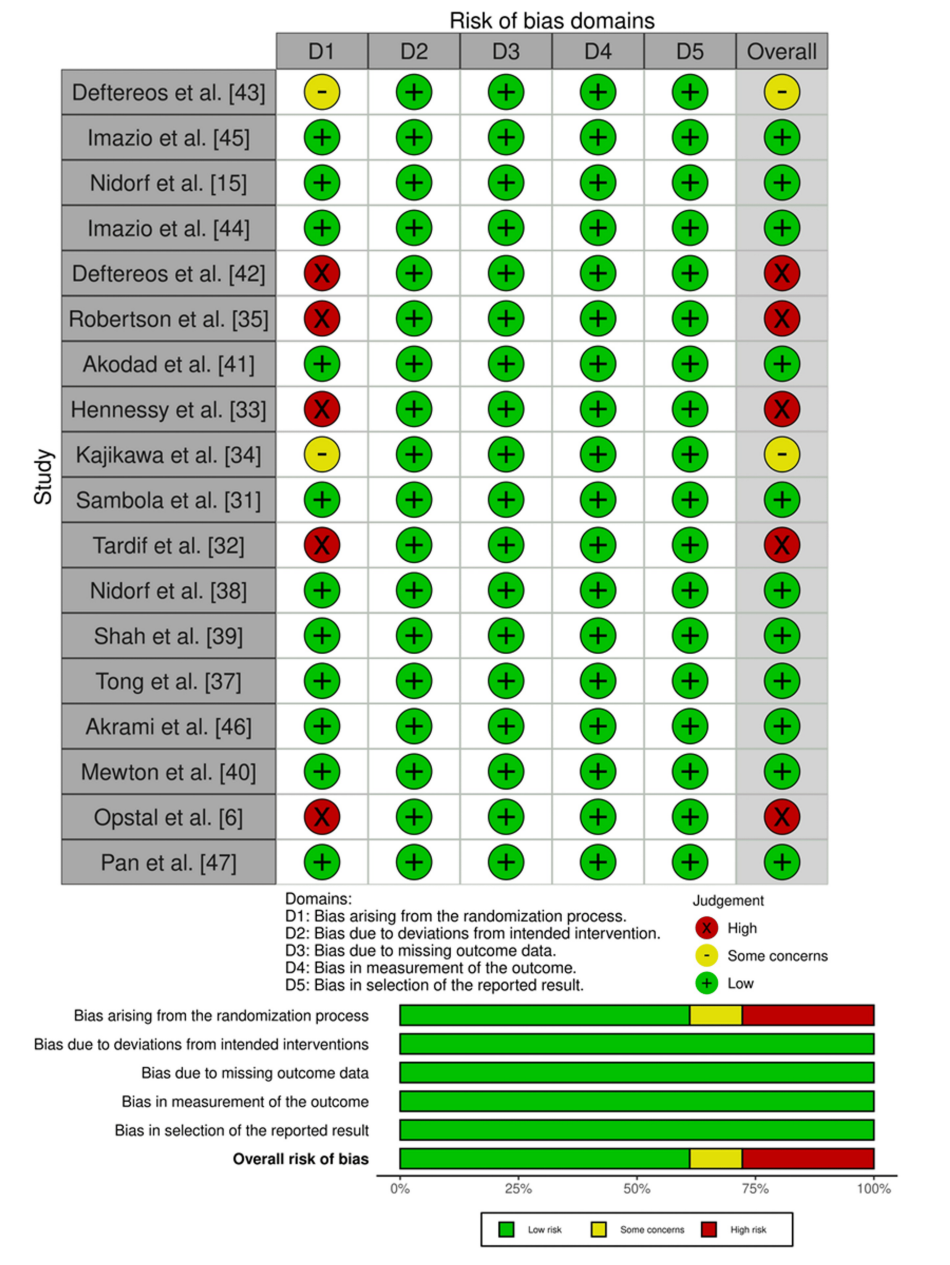
Methodological quality assessment of randomized controlled trials (RCTs).

Most of the studies (11, 61.11%) had low RoB, and two (11.11%) had some concerns in the domain of the randomization process [34,43], as these studies mentioned the method used for the randomization but did not explain the method. Meanwhile, five (27.77%) studies had high RoB in the domain of the randomization process [6,32,33,35,42], as indicated in Figure 6. These studies had a high RoB because they did not mention the randomization method nor provide any explanation for it. One observational study was evaluated using the ROBINS-I assessment tool and found to have low RoB [36].

Certainty of Evidence

The outcomes, like comorbidities among the participants in intervention and control groups, were rated with moderate certainty due to the presence of low to high RoB, even though the studies had low heterogeneity and no publication bias. Outcomes, like MACE versus all complications and mortality associated with CVD versus all-cause mortality, had very low certainty of evidence due to serious and moderate inconsistency (Table 6).

**Table 6:** Certainty of evidence using the GRADE framework. GRADE: Grading of Recommendations, Assessment, Development and Evaluation

Outcomes	Studies	RoB	Inconsistency	Indirectness	Imprecision	Publication bias	OR (95% CI)	Certainty of evidence
Comorbidities	14	Low to high	Not serious (I^2^ = 0%)	Not serious	Not serious	No	0.97 (0.92-1.02)	Moderate ƟƟƟ
MACE vs. all complications	10	Low to high	Serious (I^2 ^= 63%)	Not serious	Not serious	No	1.26 (0.92-1.72)	Very low Ɵ
Mortality-associated with CVDs vs. all-cause mortality	6	Low to high	Moderate (I^2^ = 40%)	Not serious	Not serious	No	0.89 (0.70-1.12)	Low ƟƟ

Discussion

In the present systematic review, the impact of colchicine for the treatment of CVDs has been demonstrated, extending beyond its traditional use in gout. Indeed, colchicine administration as a first line of treatment in the acute and recurrent pericarditis and gout is recommended by the European Society of Cardiology Guidelines [48]. Furthermore, it gained attention due to its anti-inflammatory properties, which directly affect the pathways and inhibit necessary actions, which have a significant impact on the heart. Overall, the current systematic review and meta-analysis concluded that colchicine administration can be helpful in reducing the inflammatory and other biomarkers. However, meta-analysis indicated a non-significant difference in comorbidities associated with the administration of colchicine, MACEs, CVD-associated mortality, and all-cause mortality. Meanwhile, a significant difference was observed in the development of the risk of all complications.

In the present study, most of the studies used colchicine with standard or conventional care; however, certain studies used colchicine as a monotherapy for the treatment of CVDs at a low dose of 0.5-1 milligram (mg)/day. Our findings are aligned with other studies, which also recommend 0.5-1 mg/day and doses exceeding 0.8 mg/kg) can cause death, while toxic doses occur from 6 to 8 mg/day [1]. The administration of colchicine at a 0.5 mg/day dose was found effective with 0.86 RR (95% CI, 0.75-0.99, while for 30 days, its efficacy was 0.86 RR (95% CI, 0.75-1.00), and for 90 days, it was 0.65 RR (95% CI, 0.46-0.92) [49]. Likewise, an umbrella review included outcomes from 48 systematic reviews of RCTs and demonstrated that colchicine exhibited efficacy in the management of acute coronary syndromes (0.72 OR, 95% CI, 0.58-0.89) and coronary heart disease (0.73 RR, 95% CI, 0.64-0.83) [50]. Administration of colchicine at higher doses for prolonged periods of time may lead to neuromuscular toxicity, myelosuppression, dermatologic issues, and liver damage [51]. Even though a lower dose of 0.5 mg/day is recommended to avoid intolerance, this dose does not even sustain serum levels beyond the threshold levels [18]. 

Furthermore, numerous studies in the present study have reported that colchicine successfully benefits in reducing the inflammatory and other biomarkers, such as CRP, IL-6, and IL-1β in patients with CVDs. It is the fact that elevated inflammatory biomarkers, including IL-6, were significantly associated with CVD, like myocardial infarction, peripheral artery disease, and heart failure. IL-1β levels were associated with worse clinical outcomes in heart failure and CAD, and elevated levels of these biomarkers were significantly associated with increased risk of CVDs [52]. A recent meta-analysis revealed that the colchicine group demonstrated a significant (p = 0.001) reduction in hs-CRP (-4.25) than in the control group, while a non-significant impact of the level of IL-1β and IL-18 [53]. Another study included 278 patients and treated them with colchicine and compared it with a control group. A significant (p<0.001) reduction in the inflammatory biomarkers was observed in the colchicine group, with the ratio of change 0.72 and 0.60 for IL-6 and CRP [54]. Moreover, 138 patients with chronic CAD were treated with colchicine, and observations were recorded at baseline and after 30 days of exposure. Inflammatory biomarkers, like hs-CRP, significantly (p<0.01) decreased to 4.40 mg/litre (L) to 2.33 mg/L, and similarly IL-6 was also significantly reduced to 2.22 nanogram (ng)/L from 2.51 ng/L [55]. Furthermore, another systematic review with 13 RCTs and 24900 patients also demonstrated that colchicine significantly reduced the risk of ischemic stroke (0.5 RR, 95% CI, 0.72-0.99) in established atherosclerotic CVDs [49]. By contrast, an RCT included 44 patients and treated with colchicine for inflammation demonstrated a non-significant (p = 0.36) difference in the mean CRP peak value among patients treated with colchicine (26.99 mg/L) and conventional treatment (24.99 mg/L) [41]. This variability among studies can be attributed to certain factors, such as study designs, as both in vitro and in vivo studies were compared, patients’ characteristics, dose and duration of the use of colchicine, timing of biomarker measurement, and baseline inflammation level. Likewise, the type of CVDs is another important factor that might affect the inflammation level. Other factors included the condition, severity level of disease, follow-up, adherence variation, supporting therapies, and laboratory methodology used for measuring the biomarkers can also contribute to the inconsistency of the outcomes across different studies.

In the present meta-analysis, outcomes indicated that colchicine administration was non-significantly (p > 0.05) associated with the presence or risk of common cardiovascular comorbidities, like diabetes, HTN, or dyslipidemia. Similar outcomes were demonstrated by another meta-analysis, which reported that colchicine was not associated with HTN and diabetes [56].

The pooled analysis indicates that colchicine was non-significantly (p > 0.05) associated with MACEs, while significantly (p < 0.05) associated with an increased risk of all complications. This may be due to the short duration of therapy and follow-up performed in most of the included studies, which mostly reported early complications. Similar findings were observed in another RCT, which included 7,062 AMI patients from 14 countries, and MACEs occurred in 322 patients in the colchicine group and 327 patients in the control group over the three years of follow-up, with 0.99, HR, 95% CI, 0.5-1.16, p = 0.93 [57]. Furthermore, 23% and 31% reduction can be observed in the MACE in recent myocardial infarction and stable atherosclerosis patients, when low-dose (0.5 mg/day) colchicine is incorporated in the guideline-based medical care for CAD patients [13]. In addition, a meta-analysis demonstrated that after the administration of colchicine, 17% and 23% reduction in the incidence of MACE (0.83, 95% CI, 0.73-0.95, p = 0.006) and eMACE (0.77, 0.63-0.94, p = 0.01) was observed. This decrease in the incidence rate was driven by the lower rate of coronary revascularization (0.73, 0.55-0.97, p = 0.03), myocardial infarction (0.78, 0.63-0.95, p = 0.02), and fewer cases of stroke in the colchicine group (0.81, 0.63-1.04, p = 0.11) [58]. A question can arise regarding the mechanism behind the reduction of MACE in CVD patients after administration of colchicine, and a possible explanation can be that colchicine has some potent anti-inflammatory properties. Chronic inflammation plays a critical role in the development and progression of plaque instability, atherosclerosis, and thrombosis, which are considered key contributors to MACEs. Colchicine inhibits microtubule polymerization, which suppresses activation of the NLRP3 inflammasome and reduces the production of pro-inflammatory cytokines, such as IL-1β and IL-6. This dampening of the inflammatory response stabilizes atherosclerotic plaques and decreases vascular inflammation, thereby lowering the risk of CVDs.

The pooled analysis indicates that colchicine was non-significantly (p>0.05) associated with CVD-associated mortality and all-cause mortality. Our findings are aligned with another meta-analysis, which also observed a non-significant (p=0.17) effect of colchicine on the CVD-associated mortality when compared with control (0.77, RR, 95% CI, 0.53-1.12) and also observed a non-significant (p = 0.05) association with all-cause mortality (1.38, RR, 95% CI, 1-1.90) [56]. Similarly, a meta-analytic outcomes indicated non-significant impact of colchicine on all-cause mortality (1 RR, 95% CI, 0.72-1.39), incidence of stroke (0.45 RR, 95% CI, 0.17-1.19), incidence of cardiac arrest (0.81 RR, 95% CI, 0.33-1.95), and incidence of recurrent MI (0.78 RR, 95% CI, 0.57-1.06) [59]. Similarly, colchicine administration was non-significantly (p = 0.49) associated with increased all-cause mortality (1.09 RR, 95% CI, 0.85-1.40); however, its administration was not associated with any cardiovascular mortality (0.90 RR, 95% CI, 0.65-1.29) [60].

Overall, clinical trials indicated that colchicine effectively reduced CVDs by 23% among patients with MI and 31% with chronic coronary disease [61]. In fact, evidence from clinical trials suggested that a low dose of colchicine effectively reduces the risk of ischemic events in CAD patients, particularly new myocardial infarction, revascularization, and strokes [62]. In addition, there are other landmark clinical trials and major cardiovascular societies, which supported the effectiveness and safety of low-dose colchicine in reducing MACE, particularly when combined with other standard anti-inflammatory therapies [63].

Strengths and Limitations

Among the strengths of this review, we employed standard PRISMA guidelines, including a reproducible and transparent literature search strategy, a clearly defined PICO framework used for the inclusion and exclusion of the studies, a clear plan for the data analysis, methodological quality assessment, and outcomes were further validated by using GRADE certainty of evidence framework. In addition, this review provides a comprehensive synthesis of current evidence on the impact of colchicine on patients with CVDs, highlights its anti-inflammatory role, and also provides insights into its clinical outcomes. It included the most relevant RCTs and most cited studies, enhancing the robustness of the study. However, certain limitations should also be considered, like heterogeneity in the study design, methodologies, populations, short follow-up period, and dose may confound the interpretation of the outcomes. Moreover, certain outcomes of the present review had moderate to high heterogeneity, which further limits the interpretation of the outcomes. Due to the unavailability of uniform data regarding the dosage and duration of usage, unable to perform subgroup analysis.

## Conclusions

This study provides insights into the evolving and increasingly significant role of colchicine in managing CVDs, underscoring its potential as an easily accessible and effective adjunctive therapy for reducing inflammatory risk beyond traditional pharmacological strategies. The findings consistently support that colchicine, primarily through its anti-inflammatory effects mediated by the inhibition of IL-6, IL-1β, and CRP, can lead to meaningful reductions in MACE. Overall, a low dose of 0.5 mg/daily of colchicine was used. Meta-analysis outcomes indicated that colchicine use was non-significantly associated with the presence or risk of common cardiovascular comorbidities (diabetes, HTN, or dyslipidemia). The pooled analysis further indicates that colchicine was non-significantly associated with MACE, CVD-associated mortality, and all-cause mortality, while significantly associated with an increased risk of all complications. Future large-scale, long-term, and diverse clinical trials are essential to confirm its safety and efficacy across broader patient populations, clarify optimal dosing strategies, and determine its role relative to newer agents in the anti-inflammatory armamentarium. Until then, clinicians should consider colchicine as part of a comprehensive, individualized cardiovascular risk reduction plan, with careful attention to patient selection, comorbidities, and potential drug interactions.
